# How do clinicians navigate end-of-life care with NIV/CPAP? A thematic analysis study

**DOI:** 10.1136/bmjopen-2025-111989

**Published:** 2025-12-23

**Authors:** David Wenzel, Lucy Bleazard, Eleanor Wilson, Jennifer Creese, Christina Faull

**Affiliations:** 1 School of Medical Sciences, Public Health and Epidemiology, University of Leicester, Leicester, UK; 2 Palliative Care, LOROS Hospice, Leicester, UK; 3 School of Health Sciences, University of Nottingham, Nottingham, UK; 4 LOROS Hospice, University of Leicester, Leicester, UK

**Keywords:** PALLIATIVE CARE, Ventilators, Mechanical, RESPIRATORY MEDICINE (see Thoracic Medicine)

## Abstract

**Abstract:**

**Objectives:**

To explore barriers and facilitators to a good death in patients with respiratory disease when advanced respiratory support, including non-invasive ventilation (NIV) and continuous positive airway pressure (CPAP), is used. Specifically, we examined healthcare professionals’ perspectives on what constitutes a good death in this context, how treatment failure is recognised, how decisions to continue or withdraw therapy are made, and the impact of providing this care on staff.

**Design:**

Qualitative study using semistructured interviews and reflexive thematic analysis.

**Setting:**

Secondary care services in a large UK National Health Service Trust, including acute medicine, general medicine, respiratory medicine and palliative care.

**Participants:**

25 healthcare professionals (19 female, 6 male) from multidisciplinary backgrounds, including doctors, nurses and physiotherapists. Participants self-identified as experienced in the provision of NIV/CPAP at the end of life. Staff working primarily in intensive care units were excluded.

**Interventions:**

None.

**Outcome measures:**

Not applicable.

**Results:**

Healthcare professionals described the complexity of caring for patients dying while receiving or recently withdrawn from NIV/CPAP. Five interrelated themes were identified: beliefs around dying well, symptom management during active treatment, recognition of treatment failure, negotiated decision-making and the process of withdrawal. Staff reported tensions between providing active treatment and ensuring comfort, inconsistent practices regarding symptom control and withdrawal, and conflicts within multidisciplinary teams. Nurses highlighted hidden psychological and relational labour in supporting patients, while doctors often described delays in decision-making to align families with treatment withdrawal.

**Conclusions:**

Caring for patients using NIV/CPAP at the end of life presents ethical, clinical and emotional challenges for staff, patients and families. Variation in practices and perspectives highlights the need for structured training, interdisciplinary approaches and greater recognition of the often hidden relational and emotional labour involved in this work, particularly among nursing colleagues. Further research should evaluate strategies to support consistent and compassionate withdrawal practices.

**Strengths and limitations of this study**

- This qualitative study provides an in-depth exploration of multidisciplinary healthcare professionals’ perspectives on end-of-life care for patients using non-invasive ventilation/continuous positive airway pressure outside intensive care settings.
- Reflexive thematic analysis offered a flexible and transparent method for identifying patterns of meaning across a diverse dataset.
- Reflexivity was explicitly incorporated through researcher diaries and supervisory discussions, strengthening the trustworthiness of the analysis.
- Recruitment was conducted within a single UK NHS Trust, which may limit the transferability of findings to other healthcare contexts.
- Although participants represented multiple professions, the sample was predominantly White and female, which may have restricted the diversity of perspectives.

## Background

 Tight-fitting ventilatory support masks including continuous positive airway pressure (CPAP) and bilevel positive airway pressure/non-invasive ventilation (NIV) are commonly used to manage respiratory failure. NIV/CPAP delivers pressurised air and supplemental oxygen through a tight-fitting mask or hood and is used for a variety of conditions with significant mortality rates.

Chronic obstructive pulmonary disease (COPD) is the most frequent indication for NIV, affecting over 2% of the UK population and 4.5% of those over 40 years of age.^1^ These patients are admitted frequently, accounting for 115 000 admissions annually with a mortality rate of approximately 16.8%.^2^ Other conditions requiring NIV/CPAP include obesity hypoventilation syndrome with an inpatient mortality rate of 15%,^3^ COVID-19 with mortality rates up to 90% in older populations outside of intensive care requiring CPAP,^4^ congestive cardiac failure (if requiring NIV/CPAP) has a mortality rate of ~13%,^5 6 6^ and neuromuscular disorders (most notably motor neuron disease).

These two treatments provide physiologically distinct interventions. However, the patient experience of suffering from respiratory failure and being treated with a tight-fitting mask that delivers pressurised oxygen demonstrates a commonality that calls for a joint assessment of their impact on care.

Despite the use of NIV/CPAP, many patients with these conditions will not survive acute admissions, but little is known about their end-of-life experience.^7^

The techniques staff use to care for these dying patients are also under-researched, despite an understanding that the experience level of nursing staff significantly influences treatment tolerance.^8^ Care can have a substantial impact on staff, and early work within this study was published that exclusively examines the impact on staff of providing NIV/CPAP care during the COVID-19 pandemic.^9^ The psychological impact on staff arises from the distress experienced by patients and their families in what is seen as an intensively medicalised dying process.

Recent work has begun to describe how NIV and related technologies shape end-of-life trajectories, including palliative use of NIV in COPD, scoping reviews of NIV in palliative care, and experiences of withdrawing assisted ventilation in motor neuron disease. However, most existing studies focus on patient and family perspectives, specific diagnostic groups, or home ventilation services, and there is limited evidence about how ward-based clinicians outside intensive care navigate end-of-life care when NIV/CPAP are used as a ceiling of treatment.^710^,^12^ Little is known about how they recognise treatment failure, balance survival and comfort, negotiate decisions with patients and families, and manage the ethical, clinical and emotional impact of withdrawing non-invasive respiratory support in acute hospital settings. This study addresses this gap by exploring multidisciplinary clinicians’ perspectives on barriers and facilitators to a good death for patients dying while receiving, or recently withdrawn from, NIV/CPAP outside intensive care.

### Aims

The aim of this study was to address the current lack of evidence on how ward-based clinicians navigate end-of-life care when NIV/CPAP are used as a ceiling of treatment. Specifically, we sought to explore: (1) how clinicians conceptualise a ‘good death’ in this context; (2) how they recognise when NIV/CPAP is unlikely to succeed; (3) how decisions are made to continue or withdraw therapy; (4) how they experience the emotional and ethical impact of providing this care and (5) how the process of withdrawal is enacted in practice.

## Methods

This study is reported in accordance with the Standards for Reporting Qualitative Research checklist, which outlines best practice for transparency across study design, researcher reflexivity, data collection, analysis and presentation of findings.^13^

### Research paradigm

We adopted an epistemological position of social constructivism, recognising that knowledge is generated through the interplay between lived experience and social meaning. Both the recurrence of experiences and their perceived significance informed theme development.^14^

### Researcher characteristics and reflexivity

The lead author (DW), a palliative care specialty registrar with extensive clinical experience of ventilatory support, conducted most interviews and analysis while maintaining a reflexivity diary that documented descriptive, interpretive and reflective notes. These were discussed with the senior author (CF) throughout data collection and analysis. A medical trainee (LB), without clinical experience of ventilatory support, contributed to data collection and initial coding. This contrast in experience enabled ‘sense-checking’ of interpretations. The approach to reflexivity was informed by Lichterman’s model of interpretive reflexivity, which highlights the researcher–participant dynamic.^15^

### Context

Participants were recruited from a single National Health Service Trust in central England, across acute medicine, general medicine, respiratory medicine and palliative care. Multidisciplinary staff were purposively sampled, including doctors of all grades, nurses and physiotherapists. Intensive care staff were excluded, as the study focused on patients more likely to be conscious rather than invasively ventilated.

### Sampling strategy

Recruitment initially used criterion sampling via Trust-wide email invitation. Further purposive sampling was employed to maximise diversity of professional backgrounds and perspectives. Participants were asked to self-identify as experienced in NIV/CPAP care; their level of experience was explored during interviews. To capture variation, staff with limited COVID-19 experience were purposively recruited later in the study.

### Data collection

Between 2021 and 2024, 25 healthcare professionals participated in individual (n=22) or group (n=3) interviews, lasting 45–90 min. Participants chose whether to attend online or in person. Interviews were audio-recorded with encrypted devices and followed a semistructured topic guide, refined iteratively as analysis progressed. Early versions and later revisions of the guide are included in the online supplemental materials.

### Data collection instruments and technologies

Interviews were transcribed verbatim by DW and LB, anonymised and coded with participant identifiers. Identifying details were redacted. Transcripts were managed in NVivo (V.14) with supplementary use of Microsoft 365 for framework organisation.

### Data analysis

We conducted reflexive thematic analysis (RTA) following Braun and Clarke’s six-phase approach and their contemporary guidance on quality practice.^16^ RTA was selected for its alignment with our constructivist epistemology and its suitability for exploring how clinicians make sense of complex end-of-life practices. Semistructured interviews provided rich, interpretive accounts consistent with this analytic approach.

Coding was inductive and flexible. DW coded all transcripts, with early transcripts also independently coded by LB to support analytic breadth rather than to seek consensus. We did not pursue inter-rater reliability, data saturation or codebook stability, as these are not features of RTA. A framework matrix was used solely as a data management tool to support immersion and familiarisation, not as a coding frame^17^ .

Themes were developed iteratively through close reading, reflexive engagement and ongoing discussion within the research team. Themes were conceptualised as patterned meanings underpinned by central organising concepts. Our analytic decisions were reviewed using Braun and Clarke’s 20-question tool.^18^

The end point of our study occurred when sufficient information power was gathered to address the research aims.^19^

### Techniques to enhance trustworthiness

Rigour was supported through purposive sampling, team-based coding and analysis, reflexive practice and supervision. Member checking was incorporated during interviews through paraphrasing and clarification. Regular author discussions enhanced credibility and dependability of interpretation.

### Patient and public involvement

Members of the LOROS Hospice Patient and Public Involvement (PPI) group contributed to study design and commented on the acceptability of aims and topic guides. Non-participant staff reviewed topic guides to enhance clarity and relevance. PPI contributors were not involved in data analysis or dissemination.

### Findings

A total of 25 participants were interviewed across 16 online-platform interviews, six in-person interviews and one group interview. This included four ward nurses, one specialist respiratory nurse, five palliative care specialist nurses, three acute medicine consultants, three respiratory consultants, three palliative care consultants, three senior house officer (resident) doctors, one registrar and two physiotherapists. The origins of quotes are attributed in the body of the text, but an overview of quote source can be found in table 1.

**Table 1:** Origins of participant quotes

Participant	Beliefs around dying well	Symptom management during active treatment	Recognition of treatment failure	Negotiated decision making	The process of withdrawal
Ward Nurse 1	X		X		X
Ward Nurse 2	X	X			
Ward Nurse 3				X	X
Ward Nurse 4				X	
Medical Consultant 1	X			X	
Medical Consultant 2		X		X	
Medical Consultant 3				X	
Palliative Care Consultant 1			X	X	
Palliative Care Consultant 2					X
Palliative Care Consultant 3					
Senior House Officer 1	X				
Senior House Officer 2					X
Senior House Officer 3	X				
Physiotherapist 1	X				X
Physiotherapist 2	X				
Palliative Care Specialist Nurse 1	X	X			X
Palliative Care Specialist Nurse 2		X	X		
Palliative Care Specialist Nurse 3			X		
Palliative Care Specialist Nurse 4				X	
Palliative Care Specialist Nurse 5	X				
Respiratory Consultant 1		X	X		X
Respiratory Consultant 2	X				
Respiratory Consultant 3			X	X	
Specialist Respiratory Nurse 1				X	
Medical Registrar 1	X		X		

The data from online versus in person interviews were similarly rich, in keeping with published literature in this area.^20^ Participants in both the group and one-to-one interviews described similar patterns of experience and emphasis in their accounts.

Participants consistently expressed, across all interviews, the intricate and complex nature of this care, extensively discussing the concept of trying to provide a good death for their patients. The fine balancing act between supportive and active care and the high symptom burden that patients experience drives a significant distress that is experienced by both families and staff. Our analysis is presented here as five themes; beliefs around dying well, symptom management, negotiated decision-making, recognition of treatment failure and the process of withdrawal.

### Beliefs around dying well

Central to this value system was a desire to provide, above all else, a comfortable and peaceful death. Comfort equated in this instance to being free from symptoms and looking externally ‘calm’. The length of time before death that a patient needed to be comfortable in order to describe the death as ‘good’ was highly variable but often cited as ‘hours’ or for the period following mask withdrawal. Family presence, either in person or via video calling, was often cited as an important part of this peaceful final moment.

You have to plan everything to do with it, to ensure that the patient has the best outcome, which would be a comfortable death

Palliative Care Nurse 1

I think the actual death that she had was a good one. It was peaceful, it was well planned, it was in an environment where she felt safe and secure and her family were with her

Physiotherapist 1

The actual death itself and that last hour, I think, was very comfortable

Senior House Officer 1

However, there was less consensus about whether a good death could be provided with the interface in situ, or whether it must always be withdrawn.

The general policy should be that it’s not appropriate or acceptable that someone dies with an NIV or CPAP mask on

Medical Registrar 1

I’d argue if the patient isn’t distressed it might be appropriate to keep the mask on

Ward Nurse 1

Where participants felt strongly that masks should be removed at the end of life, this was often informed by their own feelings towards the mask interfaces, but couched within ineffable concepts, like dignity, around the patient’s death.

Keeping it on is going to be not very comfortable for them. It’s going to be a lot of pressure. It’s not going to be a dignified death

Medical Consultant 1

Apart from the futility… It’s an undignified manner in which someone dies

Medical Registrar 1

I wouldn’t want to be on [NIV or CPAP] on the last day of my life, so why would someone else?

Ward Nurse 2

Only a few participants described situations in which dying with the mask in situ was compatible with a good death, but where they expressed this, they often cited patient autonomy as an important part of what allowed ‘on mask’ deaths to be ‘good deaths’. Patients sometimes expressed a desire to continue therapy up to the point of their death. This decision was often expressed as part of the acute illness, and no patients discussed during this study had pre-existing advance care plans.

The desire to withdraw masks at the end of life was often held in tension with a drive to help patients survive. Many participants reported that knowing that they had tried to help the patient live was an important component of achieving a good death. Sometimes this was expressed through the lens of assumed family perception of medical care, rather than from the staff perspective.

There was a man where they wrote on the RESPECT form, ‘daughter states would not tolerate NIV’, but I thought that we should try

Palliative Care Nurse 5

I think a lot of families appreciate visible signs that the medical team are doing absolutely everything, everything they can, to make the patient better. And there will be an understanding that effort was made if it was unsuccessful

Respiratory Consultant 2

Further complicating the tension between survival and withdrawal were considerations that dying may be prolonged by NIV/CPAP if not withdrawn early enough.

We were keeping her alive in a relatively uncomfortable state for a prolonged period of time. Despite all the evidence showing that she probably wasn’t going to survive

Physiotherapist 2

What we were doing by leaving the CPAP on for him wasn’t helping his breathlessness. It wasn’t helping with his survival, and we were likely prolonging his death and suffering

Senior House officer 3

### Symptom management during active treatment

When discussing patients who had died, many participants reflected on the early stages of the patient journey and the significant symptom burden that came with starting therapy and with their underlying critical illness. Some participants described patients experiencing symptomatic relief from the mask itself, but this was highly variable from patient to patient and changed over time (ie, some might find relief to start but later find it burdensome). Staff described a wide range of symptom control techniques that were used while the patient was still being treated with curative intent. Some symptom control treatments were overt in nature and were described in a similar fashion by almost all participants. Most often, this revolved around drug therapy, with the use of morphine and midazolam being the most frequently cited. Some participants described not using any drug therapy while the patient was still for ‘active’ treatment, while others reported using these medications alongside ‘active’ treatment.

We’ve started using syringe drivers so much more… low dose morphine and midazolam… obviously for COVID patients we started doing that quite early on… but now sometimes with some COPD patients I've started using it if they are actively distressed or really struggling from breathlessness… I'm a lot more liberal with syringe drivers now

Respiratory Consultant 1

Symptom control medications were most often given via the subcutaneous route to minimise the time off the mask that would be necessitated by oral medication.

Other overt forms of symptom control involved the adjustment of machine settings, adjusting the mask fit and rotation to use a hood interface instead of a mask. These adjustments aimed to minimise the tightness, discomfort and pressure sores that were caused by the interfaces.

These symptom control techniques were overt insofar as all participants were aware of them and they are easily seen as a labour to improve patient experience.

Nursing participants described a more covert form of symptom control which was less understood by non-nursing participants. This included psychological support for the patients, which at times was quite intense, and a stepwise initiation process to help patients adapt to the mask interface, including slow exposure to the pressure and a detailed explanation of what it might feel like. Patients were given agency over treatment through reassurance they could ask for breaks to eat, drink and generally rest. These mechanisms of symptom control were not explicitly described by any doctors.

I just sat with him and held his hand really. I just tried to reassure him that I wasn’t going to leave until he was settled

Palliative Care Nurse 1

It was me supporting him with his nutritional intake and also his emotional [needs], like constantly reassuring him. I know that he was a religious man… so we would put religious mantras on in the background.

Ward Nurse 2

Nurses described psychological interventions to manage many of the intrinsic burdens from NIV/CPAP, such as claustrophobia, communication difficulties due to the noise of the machine, loss of independence, increased dependency on the mask and the feeling resulting from the pressure of the air.

Mouth care was challenging to provide to all patients using NIV/CPAP. Not only is the interface a physical barrier to adequate mouth care, but the patient’s growing dependency on NIV/CPAP to breathe comfortably means it is difficult for staff to remove the mask for even a few seconds in severe disease.

He didn’t even want mouth care as he was just so breathless and anxious when it was taken off

Palliative Care Nurse 2

Participants did not describe any specific techniques for providing mouth care, other than emphasising the importance of carrying it out quickly. Some of the nursing participants described the value of care plans in ensuring that mouth care was delivered in a situation where many people find it difficult to do so.

A further aspect of NIV/CPAP use that participants reported as challenging was the false hope that the interface provided.

The difficulty was that her family had seen her be in extremis when she first came in. They had seen that there had been some improvement initially… I think that they struggled to accept that actually yes the [CPAP] was keeping her alive but her prognosis was very poor and in reality she was getting worse by the day, not better

Medical Consultant 2

Participants reported multiple incidents of struggling to manage this false hope and reflected a frequent feeling that conversations at the outset of treatment had inadequately prepared the patient for the reality they may not survive.

I suspect what they’re [medical registrars] saying is, ‘you’re very unwell, we need to put you on this breathing machine’ and I think that’s probably where the conversation stops essentially

Medical Consultant 2

The false hope was seen as an integral part of the burden of therapy but often lamented in the context of how it complicated explanation to both patients and families that treatment had failed.

Participants did not describe using any formal symptom assessment scales as part of their routine care for patients even on direct questioning, instead relying on a gestalt assessment of patient well-being.

### Recognition of treatment failure

Treatment was primarily regarded as an active therapy used to increase the chance of a patient’s survival, but it was also regarded as a symptom control tool and a way of ‘buying time’ even if death felt inevitable.

It worked to help with her breathlessness and bought some time for [the family] to reflect on the fact she was going to die

Palliative Care Consultant 1

This is for an incredibly oxygen dependent patient [starting CPAP]—might just buy them a bit more time for easier communication

Respiratory Consultant 3

Participants described a range of ways in which they assessed when death was inevitable. In response to the question, ‘How did you know the patient was dying?’, many spoke about drawing on bedside observations (such as oxygen saturations and respiratory rate) alongside biochemical indicators (including inflammatory markers and serum oxygen concentration). Some participants described recognising a greater dependency on the mask as a sign of treatment failure. Others struggled to articulate exactly how they knew, instead framing it as an intangible aspect of clinical judgement.

Sometimes it’s just that you know by looking at them. A change in how they look. A change in their observations. Bloods. Their increased need for [NIV]. Sometimes you just know, don’t you?

Palliative Care Nurse 2

Many participants also described a slow or absent improvement on therapy as an indication of its inevitable failure, but how long constituted ‘slow’ was variable among clinicians and at times cited as a source of conflict.

Don’t give up too soon, don’t have the 48 hour window that the [other team] have. Keep them going for a couple of weeks because we have had people leave with no oxygen [after prolonged treatment]

Respiratory Consultant 1

I was involved in their care about 48 hours after they had been admitted. By this point they weren't improving on [NIV]. The lady was unfortunately, not going to survive this hospital admission so we had to discuss end of life care.

Medical Registrar 1

it feels like there’s lots of people who are on it for days or a week, and I started to use the term ‘stably sick’… nothing was really changing, or getting worse, [or] getting better either and it felt like when it got to that point it became harder for the team to make the decision to stop”

Palliative Care Nurse 3

This intangible transition in thinking from ‘could survive’ to ‘inevitably dying’ often happened at different times between different professionals looking after the same patients.

I think it was recognised by the medical team and the senior nurses before myself… actually he wasn’t responding and he was becoming worse—less responsive, less alert, bloods still deranged despite antibiotic treatment

Ward Nurse 1

This deeply subjective opinion on when death is ‘inevitable’ contributed to the tensions between staff members and even between staff and patient families (explored further below). There was a dichotomy in the opinion of NIV/CPAP: it is either working by improving the illness, symptoms or buying time, or it is not working because of a failure to improve symptoms, biochemical parameters or because no further time is required. However, determining when this happens is obscured within complex clinical reasoning and is not represented by a single point in time; rather, NIV/CPAP transitions from ‘working’ to ‘not working’ at different rates for different individual staff.

### Negotiated decision making

As explored above, NIV/CPAP have clear goals: improve the illness, improve symptoms and buy time. However, there is subjectivity around how long treatment should continue until improving the illness is no longer possible, and to what extent it is improving symptoms. Thus, decision-making around the withdrawal of NIV/CPAP was extremely complex and influenced by many factors outside individual clinicians’ control.

Weighed most highly in this decision-making were patients’ wishes, and where an absolute opinion was given, staff were often happy to support this; in many ways, exempting them from the complex clinical reasoning that would otherwise be required.

It ended up being on a night shift that she just got to the point where she said ‘enough is enough’ and took the hood off and quite shortly after that she passed away

Specialist Respiratory Nurse 1

Patients may be presented with different clinical approaches and supported to choose their own treatment, allowing them to express their feelings on the efficacy of symptom control.

When we’re talking about the latter stage… we’ll give you the form of oxygen that makes you comfortable… and saying, you know, ‘as we’re aiming for comfort it will be what form is most comfortable for you’.

Respiratory Consultant 3

The wishes of family were also highly weighted, especially in situations where patients lost capacity to make decisions. Staff reported family goals were frequently aligned with their own: to prioritise the comfort of their dying loved one.

There were three family members… two of whom were very supportive of the mask coming off because they could see her [patient A] pulling at it

Palliative Care Consultant 1

However, participants provided examples of times when lack of consensus on the best course of action could cause tensions.

One of them continued to find that really difficult. And just kept asking his mum [patient A] to not give up and keep fighting and trying to live… he continued to talk about ‘I’ve made you banana cake please, just try a bit harder’

Palliative Care Consultant 1

There was a general preference to have whole family agreement before undertaking treatment withdrawal and staff were keenly aware of the responsibility they had to families.

There’s our duty to our patient but we also know how death is for a family, also impacts on how they grieve going forward

Respiratory Consultant 3

At times, getting consensus from families and ensuring the patient had transitioned from ‘could survive’ to ‘death is inevitable’ required waiting. There was a general expectation that family wishes should align with the medical team before NIV or CPAP was stopped. Frequent, complex and senior-led communication was needed to facilitate this.

I think if you just go straight in and say, ‘I think she’s dying and we’re stopping the machine’ I think [the family] would have a lot of difficulty reconciling that… I think maybe having a bit of time to think about what was happening they were more understanding that on the second day

Medical Consultant 2

If there is a disagreement with the family, then it’s providing the facts as far as we are aware of them. But also pointing out that we are giving a pointless therapy and that actually this patient is going to die and that being the case, we want to make sure we give them a dignified death

Medical Consultant 3

This deliberate delay to allow family alignment was only reported by senior decision makers. Nursing participants frequently felt that decisions in withdrawing therapy were unnecessarily delayed.

[We] knew she was dying. We knew that we weren’t probably going to get her back. And I say this with respect but, I think that sometimes it can take doctors a little bit longer.

Ward Nurse 3

Delayed decisions were additionally attributed to the gravity of the decision itself, a burden of responsibility and poor continuity of staff. Many decisions came following a multidisciplinary team (MDT) discussion and shared decision making.

Ever since COVID we do a lot more decision making jointly… that’s with our consultants, physio[therapist]s, [specialist respiratory nurses]

Palliative Care Nurse 4

Despite this, conflict was referenced frequently. On most occasions where conflict was noted, the participants made references to advocacy and assuming the voice of the patient.

We’ll throw everything at them and see what happens rather than acknowledging people do sometimes just die… it’s about giving her as dignified a death as we possible could… I will always be the patient’s advocate

Ward Nurse 3

I would definitely push for review sooner [with more experience]—if you don’t agree with this then we need to get a senior review. I would definitely be more a voice for my patient

Ward Nurse 4

Ultimately, decisions on when, or if, to withdraw NIV/CPAP resided with the most senior doctor available at that time. Conflict, therefore, not only occurred between professions, but also existed between different senior doctors.

There were shifts where I came on the nurses said ‘this patient’s dying and the consultant before you wanted to persist with the mask’… more often than not, I’d agree with the nurses

Medical Consultant 1

### The process of withdrawal

The knowledge and experience participants had around the process of withdrawal was very varied. While all participants self-identified as experienced in the provision of NIV/CPAP, the detailed knowledge of the actual process of withdrawal came only from senior doctors and the nursing team. Junior (now known as ‘resident’ doctors) were less knowledgeable:

I think I’ve seen you’re supposed to give some morphine and midazolam as you start to withdraw, I don’t know a lot about it.

Senior House Officer 2

Many staff described only some components of withdrawal, but broadly, it followed a process of preparation and planning (gathering staff, allocating roles, silencing alarms and removing telemetry), giving medications (most commonly morphine and midazolam, occasionally levomepromazine), removing the mask, applying oxygen and then staying with the patient, giving more medication as needed. It was reported that withdrawals should be done in working hours when more staff are available, if possible, due to the time it takes to withdraw NIV/CPAP while assuring good symptom control and adequately supported family members.

However, there were several areas where management was less clear with participants following different treatment processes. These included the use of continuous subcutaneous infusions of medication (CSCI), pressure weaning, sedation and the use of post-withdrawal oxygen.

Where able, participants often initiated CSCIs before withdrawal, but if the deterioration was rapid, there wasn’t always time. They were sometimes utilised post-withdrawal, if the patient survived for a prolonged amount of time.

Some participants described slowly reducing the pressure settings of NIV/CPAP prior to withdrawal, while others preferred to take the mask off straight away.

Sort of slowly wean the settings down and then eventually take it off. Or you just make sure somebody is really well sedated and then take the NIV off completely

Physiotherapist 1

The level of sedation that a patient required prior to withdrawal was also inconsistently described. Some described symptom-controlled patients being fully awake during withdrawal, while others reported patients should be sedated.

We didn’t take the mask off until she was heavily sedated and comfortable.

Physiotherapist 1

Ask them if they are ready for the mask to be removed, which most of the time they are

Ward Nurse 1

After withdrawal from NIV/CPAP, the majority of participants described applying post-withdrawal oxygen, via a diversity of devices from high flow to nasal cannula. This was offered for patient comfort in most instances, but a few participants identified that family could become distressed if oxygen is withdrawn completely.

I think the biggest reason why we might do that is because the family can sometimes get more distressed if the patient has been on 100% oxygen and then no oxygen

Respiratory Consultant 1

Variation in knowledge about withdrawal appeared to stem from participants’ perceptions that existing guidelines offered limited practical value and from the absence of structured training.

Some participants described withdrawal guidance documents, published by their hospital trust, as being helpful as an aide-mémoire for how to approach withdrawals. Others felt these were of limited value because of how personalised withdrawal practice should be.

Despite the nuances and apparent complexity of withdrawal, no participants described receiving any training to support their clinical practice. Instead, they described a system of experiential learning and trial and error to develop their own practice, even if this process was traumatic for staff.

People don’t like withdrawals… and the impact it has on them. All it takes is one bad experience [withdrawing NIV/CPAP]. Where you weren’t able to support that patient with whatever you were going to do to manage their anxiety, distress and breathlessness… withdrawals are experience-led. At least that’s the way I see them”

Palliative Care Consultant 2

Some participants described experiences of being mentored, or mentoring others, in withdrawal practice.

You don’t say, ‘well you’re new go and deal with this’. It’s very much an under my wing process

Palliative Care Consultant 2

Few participants regarded the process of withdrawal as simple, describing it as a situation where the mask could be removed without planning or preparation. In contrast, many recounted examples where this approach, taken by another member of staff, had led to a difficult experience.

I just take the mask off really… I generally take the mask off and then turn of the machine. And you just accept it’s going to alarm at you.

Ward Nurse 3

[the consultant] was happy for the mask to be removed, so the mask was removed [by a specialist nurse]. But once they removed the mask he settled for a few moments, but then very acutely, very quickly, became more distressed because he couldn’t breathe. And became massively tachypnoeic and was trying to sit up to get his breath, which was horrific.

Palliative Care Nurse 1

## Discussion

### Defining good care and its effect on decision making

The care of patients requiring NIV/CPAP is intricate, often presenting a delicate balance between the potential for survival and the risk of a heavily medicalised or prolonged death. Healthcare professionals must manage this complexity amid significant patient symptom burdens and family distress. Much of this process mirrors analogous decisions in intensive care, where life-sustaining treatment may similarly be withdrawn if shown to be ineffective. Like the intensive care experience, staff become distressed if technology is felt to have prolonged dying or intruded on the dying experience.^21^ Where experiences differ is in the patients ability to partake in decision making. In intensive care, few patients are able to take part in decisions about their care,^22 22 23^ while our participants often discussed patients who were conscious, sometimes able to communicate their wishes during the process of NIV/CPAP withdrawal.

On occasions where patients were not able to express their wishes, the views of family members were sought. Participants described supporting families to understand the medical view of treatment failure as well as deploying deliberate delays in decision making to allow time for families to understand the decision to withdraw treatment. A similar foregrounding of family experience and expectation can be seen in existing literature, but there are similar concerns over whether this represents true shared decision making when there is so often an expectation that families must adopt the medical viewpoint.^24 25^

The consequences of NIV/CPAP care may have long-reaching implications for surviving loved ones, where the manner of their loved one’s death can influence the risk of complicated grief and post-bereavement psychiatric illness.^26 27^ Being asked to partake in medical decision making, involving family in this complex care, can also increase the risk of complex grief developing.^28^

This landscape is further complicated by the absence of an agreed ‘phenotype’ of patient who is less likely to survive, that is, staff consider it challenging to predict prospectively who will die despite NIV/CPAP. This creates complex ethical challenges where staff must determine when the risk of a distressing death outweighs the chance of surviving an acute illness. This is especially complex when the repeated nature of airway disease exacerbations is considered. For example, patients with COPD are likely to suffer several exacerbations before death, and understanding, or predicting, which exacerbation is unrecoverable leads to participant uncertainty.^29 30^

When, however, it is agreed a patient is dying despite the use of NIV/CPAP, staff objectives shift to ensuring a ‘good death’; a value which is often applied retrospectively to narrate the patient’s dying journey. Consistent with existing literature,^31^ our study found that patients should be physically and emotionally comfortable, have family present and maintain dignity during death. Many staff equated a 'dignified’ death with being ‘mask-free’. The desire for a mask-free death was often linked by participants to a personal aversion to mask wearing. Since staff cannot fully comprehend or explore the patient’s experiences during the dying process, they frequently based their experiences with NIV/CPAP on the visual appearance of dying with the interface in situ. Controlling the appearance of the patient provides the ‘dignity’ essential for a good death.

The absence of evidence or policy leads to staff foregrounding their personal values, which may not be shared universally, to inform care. These actions often intuitively align with research in analogous areas of end-of-life care.^27 32^ In this way, staff act to reduce the impact of bereavement on surviving family.

This desire by staff to provide a NIV/CPAP-free death created tension among the MDT as the transition from active treatment to end-of-life care was deeply nuanced with a graduated transition in shared understanding and clinical likelihood of survival. Participants expressed that the point at which individual staff concluded that a patient was dying despite NIV/CPAP was quite diverse and this led to moral injury at perceived delays to withdrawal.^9^ Further disagreement within the MDT was driven when some staff favoured continuing therapy to maximise survival chances, relieve symptoms with NIV/CPAP or buy time, while others advocated for urgent withdrawal. This can contribute to the risk of conflict between medical teams which can exacerbate distress for patients, families and staff, and is a risk factor for poor patient care.^33^

Staff often expressed their willingness to advocate for the patient, sometimes against the management plan of decision-makers. Despite a high emphasis on patient autonomy, when patients were unable to speak, participants often subsumed the patient’s voice, representing their own values regarding NIV/CPAP withdrawal. Some senior decision-makers described alignment with this advocacy while others disregarded it, further increasing the risk of MDT conflict. This MDT conflict can be seen analogously in intensive care units where improved collaborative teaching was recommended to reduce the risk of disagreements impacting patient care.^24 34 34^

Barriers to providing a good death included the siloing of care between professions. Doctors and senior decision-makers undertook a hidden labour, working to balance their clinical judgement with the emotional care of family members and survival chances. They described a slowed decision-making process to allow family members time to align themselves with a plan to withdraw; however, the nursing team sometimes interpreted this as reluctance or inability to act. This interpretation was further compounded by wide acknowledgement of the ethically, clinically and emotionally challenging nature of withdrawal decisions. Doctors more often expressed concerns that everything to save lives should be undertaken, and some of the delayed decision making may have been related to a greater level of perceived culpability for patient death and a generalised notion that what can be done, should be done.^35 36^

The nursing team undertook their work in an apparent silo, unshared with the MDT. Typically, they described an intense one-on-one relationship with the patient providing empathetic care and psychological support. Their efforts to ease the initiation of therapy occur privately between themselves and the patient and their knowledge of physical and emotional need was often more richly described than with doctors. This work, in keeping with the hidden labour of nurses described in existing literature,^37^ is highly individualised to the patient. Understanding the scope and impact of this relational labour offers a valuable avenue for future research, particularly in how such work is supported, recognised and integrated into MDT decision-making.

### Symptom control

Our study highlighted a broad spectrum of patient responses to NIV/CPAP, ranging from those who experienced symptom relief to those whose symptoms were exacerbated by the interface. This is in keeping with existing work that explores NIV as a palliative treatment. It may provide some symptom control, but the mask interface can drive intolerance and discontinuation.^38^

Staff employed a variety of pharmacological (morphine, midazolam and continuous subcutaneous infusions) and non-pharmacological strategies (interface adjustment, breaks off NIV/CPAP and psychological support) to manage these symptoms, underscoring the necessity for a personalised approach to care. Pharmacological methods for symptom control were integrated into active care as well as after withdrawal. This approach is supported by existing literature indicating the minimal impact on mortality and morbidity from proportionate, titrated use of medications such as morphine and midazolam in conditions like stable COPD and heart failure.^39 40^ However, further research is required to explore the impact of these medications on NIV/CPAP-treated acute respiratory failure.

### The withdrawal process

Once a decision to withdraw NIV/CPAP is made, there is significant variation in knowledge and understanding of the withdrawal process among staff, leading to inconsistent practices. Some staff reported personal practices that starkly contrasted with those that other staff considered best practice. There is no consensus in the literature on best practice for NIV/CPAP withdrawal as seen in variation in regional guidelines,^41^ and there is a lack of objective measures for what constitutes a ‘good’ withdrawal. However, minimising symptom burden at the end of life is a fundamental tenet of palliative care. Palliative care specialist nurses and consultants demonstrated extensive knowledge of the withdrawal process and often intervened to lead care in challenging situations.

### Recommendations

Currently, the withdrawal of NIV/CPAP is largely learnt through observation and experiential trial, with staff gaining skills informally. This study highlights the need for preparatory training that builds on expertise already present within clinical teams, much of which, in this setting, was provided by palliative care.

While palliative care specialists are highly skilled in withdrawal, it is neither feasible nor sustainable for them to lead all such procedures given patient volume and the emotionally demanding nature of the work. Instead, we recommend that withdrawal be supervised initially by an experienced mentor until staff members develop sufficient competence to lead independently.

Holistic, interdisciplinary training programmes could minimise the need for direct palliative care involvement while promoting coordinated teamworking across the patient pathway, from initiation of NIV/CPAP through to withdrawal. Such programmes should also recognise and incorporate the often hidden labour undertaken by nurses in symptom management and patient support.

Although training cannot fully substitute lived experiential learning, it may safeguard continuity of care knowledge in high-intensity environments where staff turnover is high and improve the experience for patients, families and professionals at the end of life.

### Study limitations

This study was conducted within a single NHS Trust, which may limit transferability to other contexts. Trustworthiness was supported through reflexivity and investigator triangulation. The lead researcher (DW) maintained a reflexive journal during data collection and analysis, documenting positionality, assumptions and evolving interpretations; these were discussed regularly with a senior academic (CF) and integrated into analytic decisions. Investigator triangulation was achieved through the involvement of a multidisciplinary author team (medicine, palliative care, anthropology and research) who reviewed coding and theme development from different perspectives.

Postinterview participant validation of themes was not undertaken; instead, interviewers used paraphrasing and clarification during interviews to support accurate representation of accounts. Excluding intensive care staff ensured focus on ward-based practice, but may have overlooked perspectives from ICU where analogous challenges occur. Participants self-identified as ‘experienced’, though this varied, and we did not capture where training was received, raising the possibility that some views were shaped by local practice. Finally, the sample was predominantly female and White, with limited ethnic diversity, which may constrain perspectives on cultural and gendered aspects of end-of-life care.

## Conclusions

This study identifies the multifaceted challenges and complexities inherent in the care of patients requiring NIV/CPAP. It is evident that healthcare professionals must navigate a delicate balance between providing life-sustaining treatment and ensuring a dignified, minimally distressing end-of-life experience. The study highlights the significant variability in patient responses to NIV/CPAP, necessitating a highly individualised approach to symptom management through both pharmacological and non-pharmacological means.

Our findings reveal substantial ethical dilemmas faced by healthcare professionals, particularly concerning their subjective values around what constitutes a ‘good death’. The diversity of these values often leads to disagreements within the MDT, which risks conflict that can exacerbate the emotional distress experienced by patients, families and staff. The study also exposes the siloed nature of work in NIV/CPAP care, with distinct roles for nurses and doctors that sometimes lead to misunderstandings and perceived reluctance or delay in decision-making.

This complexity and challenge in providing good care is compounded by the lack of consistent knowledge and practices regarding the withdrawal process among staff, highlighting a critical gap in training and education.

## Supplementary material

10.1136/bmjopen-2025-111989 online supplemental file 1

10.1136/bmjopen-2025-111989 online supplemental file 2

## Data Availability

Data are available on reasonable request.
