# Supplementary material for: How do clinicians navigate end-of-life care with NIV/CPAP? A thematic analysis study
Source: BMJ Open. 2025 Dec 23;15(12):e111989. doi: 10.1136/bmjopen-2025-111989 (PMC12730799; doi:10.1136/bmjopen-2025-111989)
Supplement: online supplemental file 1 [file bmjopen-15-12-s001.docx]

**SPARS: Symptom Palliation when Non-Invasive Advanced Respiratory Support is used at end of life for treatment of respiratory failure**

**Topic Guide**

Reconfirm Consent

Before we start can I please confirm you have had a chance to read through the participant information sheet? Do you have any questions from this?

I would like to remind you at this point that your participation, while appreciated, is voluntary and you can stop the interview at any point you want. As you know to help with our data collection I will be audio recording this interview. I will turn on the audio recorder now.

Are you still happy to consent to being a part of this study as per the items in your signed consent form?

Introduction

Thank you for agreeing to participate in this interview/focus group. We are interviewing you to better understand your experiences and any challenges of providing care to dying patients who are using, or have recently used non-invasive advanced respiratory support (such as CPAP or BiPaP/NIV). There are no right or wrong answers to any of my questions, I am interested in your own experiences and thoughts.

Contextualisation
Can you please describe your role in the hospital and outline what sort of experience you have caring for patients on CPAP or NIV?

I would like you to think back to a memorable patient of yours – who received CPAP or NIV but died. Most of my questions will be about this one case, but feel free to talk about other patients as we go through.

Was your memorable patient receiving CPAP or NIV?

Why did your patient need CPAP/NIV? What were the factors that were used to make that decision?

How was the decision to start CPAP/NIV discussed with the patient/family?

How did they react to the mask therapy being started?
Prompt: did they need any medication to make the mask more tolerable?

Did the mask help them/their symptoms?

Did the patient talk to you at all about having the mask?

Did the patient talk to you at all about their illness and what was happening?

*What was your role with the patient in their last days?*

Decision Making

Was your patient recognised to be dying before they died?
*If So: Why were they thought to be dying?
If Not: How would you normally know a patient was dying? Why did that not happen in this case do you think?*

Was there a decision made to remove the patients mask?
*If So: Why?
If Not: Why not?*

How did you feel about this decision?

How did the team discuss this decision and the plan?
*Prompt: How was it discussed with the patient and their loved ones?*

Were there any disagreements over whether mask therapy should be stopped or continued?
*If Yes – What were they? How were they resolved?*

Mask removal

Who physically removed the mask?

How was the removal carried out?
Prompt: were any medications given before or after? Did the patient receive oxygen after?

Do you think it makes a difference to the patient if they die with the mask in place or removed before they die? Why?

Does it have any impact on their family?

How do you feel about it?

Symptom management

Do you recall What symptoms the patient had?

Did the patient have symptoms managed in anyway?

Communication and Mask Removal
*If mask left in place: I would like now for you to think of a patient who’s mask was removed before they died.*

Who was involved in the decision to remove the mask?
*If patient not included: why was patient not included?*

In your experience, which type of professional is normally responsible for physically taking the mask off?

Do you think this affects them? Is this something you have ever discussed with your colleagues?

Is there any sort of protocol people follow when removing NIV/CPAP? Or are plans provided by the medical team?

Do you think it is helpful to have a ‘plan’ for removing an NIV/CPAP mask? Why?

What things should be part of that plan?

Do you have an ethical concerns about the removal of a mask/NARS from a patient? Have you had conversations with colleagues about any ethical concerns?

Reflection
Did your patient have a ‘good death’
Prompts: What made it a good death?
 What would have made it a good death? What might have been done better in retrospect?

Was there anything that made achieving a good death more difficult?

To round up, can you think of one key lesson you would want people to know about taking care of patients receiving CPAP or NIV?

Thank you for your time, there are no more questions from me. However, is there anything else you felt was important to say but didn’t get a chance to?

Thanks, I’ll stop the recording now.
