# Supplementary material for: How do clinicians navigate end-of-life care with NIV/CPAP? A thematic analysis study
Source: BMJ Open. 2025 Dec 23;15(12):e111989. doi: 10.1136/bmjopen-2025-111989 (PMC12730799; doi:10.1136/bmjopen-2025-111989)
Supplement: online supplemental file 2 [file bmjopen-15-12-s002.docx]

**SPARS: Symptom Palliation when Non-Invasive Advanced Respiratory Support is used at end of life for treatment of respiratory failure**

**Topic Guide**

Introduction

Thank you for agreeing to participate in this interview/focus group. We are interviewing you to better understand your experiences and any challenges of providing care to dying patients who are using, or have recently used non-invasive advanced respiratory support (such as CPAP or BiPaP/NIV). There are no right or wrong answers to any of my questions, I am interested in your own experiences and thoughts.

Contextualisation
Can you please describe your experience of caring for patients on CPAP or NIV?

I would like you to think back to a memorable patient of yours – who received CPAP or NIV but died from ideally any condition other than COVID-19. Most of my questions will be about this one case, but feel free to talk about other patients as we go through.

Please, tell me about them.

How was the decision to start CPAP/NIV discussed with the patient/family?
Prompt – if you weren’t part of that conversation, what things would it be important for you to tell a patient under your care before starting CPAP?

How do patients normally react to starting mask therapy?

Did the mask help them/their symptoms?
Prompt: if you weren’t present when it was started, do masks usually help with these symptoms?

What, if anything, is different about nursing a patient on NARS versus a patient with a critical illness not on ventilatory support?

What made you choose this patient over any of the others that you’ve treated?

*What was your role with the patient in their last days?*

Decision Making

Was your patient recognised to be dying before they died?
*If So: Why were they thought to be dying?
If Not: How would you normally know a patient was dying? Why did that not happen in this case do you think?*

Was there a decision made to remove the patients mask?
*If So: Why?
If Not: Why not?*

How did you feel about this decision?

How did the team discuss this decision and the plan?
*Prompt: How was it discussed with the patient and their loved ones?*

Were there any disagreements over whether mask therapy should be stopped or continued?
*If Yes – What were they? How were they resolved?*

Mask removal

Who physically removed the mask?

How was the removal carried out?
Prompt: were any medications given before or after? Did the patient receive oxygen after?

Do you think it makes a difference to the patient if they die with the mask in place or removed before they die? Why?

Does it have any impact on their family?

How do you feel about it?

Symptom management

Do you recall What symptoms the patient had?

Did the patient have symptoms managed in anyway?

*If participants discuss drug use as symptom management explore the type, dose and frequency of meds they commonly use*

Does using mask therapy change the way you provide your care?
 *Prompt do you adapt what your doing to adjust to the mask?*

Communication and Mask Removal
*If mask left in place: I would like now for you to think of a patient who’s mask was removed before they died.*

Who was involved in the decision to remove the mask?
*If patient not included: why was patient not included?*

In your experience, which type of professional is normally responsible for physically taking the mask off?

Is there any sort of protocol people follow when removing NIV/CPAP? Or are plans provided by the medical team?

Do you think it is helpful to have a ‘plan’ for removing an NIV/CPAP mask? Why?

What things should be part of that plan?

Reflection
Did your patient have a ‘good death’
Prompts: What made it a good death?
 What would have made it a good death? What might have been done better in retrospect?

To round up, can you think of one key lesson you would want people to know about taking care of patients receiving CPAP or NIV?

Thanks, I’ll stop the recording now.
