# Supplementary material for: How do clinicians navigate end-of-life care with NIV/CPAP? A thematic analysis study
Source: BMJ Open. 2025 Dec 23;15(12):e111989. doi: 10.1136/bmjopen-2025-111989 (PMC12730799; doi:10.1136/bmjopen-2025-111989)
Supplement: Supplementary file 3 [file bmjopen-2025-111989.reviewer_comments.pdf]

PEER REVIEW HISTORY

BMJ Open publishes all reviews undertaken for accepted manuscripts. Reviewers are asked to complete a checklist review form and are provided with free text boxes to elaborate on their assessment. These free text comments are reproduced below.

ARTICLE DETAILS

Title (Provisional)

How do clinicians navigate end-of-life care with NIV/CPAP? A thematic analysis study

Authors

Wenzel, David; Bleazard, Lucy; Wilson, Eleanor; Faull, Christina; Creese, Jennifer

---

VERSION 1 - REVIEW

**Reviewer**	**1**
**Name**	**Jones, Kerry**
**Affiliation**	**The Open University, HWSC WELS**
**Date**	**27-Nov-2025**
**COI**	**None**

Dear Authors,

Thank you for submitting this paper for review. It is one which I have a deep interest in and I found it compelling due to the topic and also that it is a subject that is under researched. For that reason, it will be welcome addition for readers who may find that much of what they read resonates with their own experience in end-of-life care, namely the conflict in decision making, the incongruency of when and when not to withdraw treatment and the futility of policy due to a lack of evidenced guidelines. What is apparent is advocating for patient advocacy which entails a significant level of emotional labour which again is a matter that readers will recognise.

In terms of revision I have a few comments and recommendations to make:

1. It would be helpful to outline how may participants took part in the study by their profession i.e., the number who were physiotherapists/ nurses/ etc.

2. The abstract mentions (line number 49) the hidden labour of nursing colleagues. Does this not apply more widely?

3. Methods:

- good to see PPI and also staff review of the topic guide.

- Braun and Clarke (2006) RTA. As per the authors Braun and Clarke I would strongly suggest you visit their 2020 publication which adheres to a contemporary approach and to ensure that the 20 critical questions they pose have been addressed.

Findings:

Line 52 under the theme of symptom management. It seems the narrative excerpt should be 'some' instead of 'come'?

Page 11, line 12-13: No symptom assessment described. Was this not a part of the topic guide or if not why was it not further explored? To aid clarity to the sentence here.

The study limitations are noted and reflect the location and breadth of the study.

Finally, thank you for bringing professional perspectives to the fore in an end-of-life context and to the inherent complexities involved.

**Reviewer**	**2**
**Name**	**Mardani-Hamooleh, Marjan**
**Affiliation**	**Iran University of Medical Sciences**
**Date**	**01-Dec-2025**
**COI**	**None**

Change the study topic to this form:

How do clinicians navigate end-of-life care with NIV/CPAP?

A thematic analysis study

Use references from the last 5 years in the introduction and discussion.

The introduction is incomplete. Complete the review of previous studies. What was the gap in the previous literature? What was your main concern for doing this work?

The method is flawed. Report the method based on the checklist for qualitative studies. Reference this checklist and write down exactly what it assesses. All subsections of the method are flawed and this section needs serious revision.

VERSION 1 - AUTHOR RESPONSE

Reviewer: 1
Dr. Kerry Jones, The Open University
Comments to the Author:
Dear Authors,
Thank you for submitting this paper for review. It is one which I have a deep interest in and I found it compelling due to the topic and also that it is a subject that is under researched. For that reason, it will be welcome addition for readers who may find that much of what they read

resonates with their own experience in end-of-life care, namely the conflict in decision making, the incongruency of when and when not to withdraw treatment and the futility of policy due to a lack of evidenced guidelines. What is apparent is advocating for patient advocacy which entails a significant level of emotional labour which again is a matter that readers will recognise.

If appropriate, and I apologise if this oversteps bounds, but I would also like to share a related piece of work that may be of interest to you. This ethnographic study explores end-of-life care in similar clinical contexts and complements the issues raised in the present manuscript: https://pubmed.ncbi.nlm.nih.gov/41017133/ . I include this only in case it is of relevance/interest to you.

In terms of revision I have a few comments and recommendations to make:

1. It would be helpful to outline how may participants took part in the study by their profession i.e., the number who were physiotherapists/ nurses/ etc.

Thank you for this suggestion. This information was included in Table 1, but we agree that it improves clarity to present it in the text as well. We have therefore added the following sentence under Findings: This included four ward nurses, one specialist respiratory nurse, five palliative care specialist nurses, three acute medicine consultants, three respiratory consultants, 3 palliative care consultants, three senior house officer (resident) doctors, one registrar and two physiotherapists

2. The abstract mentions (line number 49) the hidden labour of nursing colleagues. Does this not apply more widely?

We agree that hidden labour is not unique to nursing. However, we wished to foreground nursing colleagues because they described particularly intense relational and emotional labour in supporting patients and families. We have therefore clarified the abstract as follows: ...and greater recognition of the often hidden relational and emotional labour involved in this work, particularly among nursing colleagues.

3. Methods:

- good to see PPI and also staff review of the topic guide.
- Braun and Clarke (2006) RTA. As per the authors Braun and Clarke I would strongly suggest you visit their 2020 publication which adheres to a contemporary approach and to ensure that the 20 critical questions they pose have been addressed.

Coincidently this exact question list was discussed in our last research meeting. I have rewritten the analysis section. This makes the 6 steps less explicit but prevents too much duplication when addressing the 20 questions. I have listed the questions below with a response for each:

1. Do the authors explain why they are using TA, even if only briefly?

Yes. We now explicitly state that reflexive thematic analysis (RTA) was chosen for its suitability in exploring clinicians' meaning-making processes around end-of-life care with NIV/CPAP, and its alignment with our constructivist epistemology.

2. Do the authors clearly specify and justify which type of TA they are using?

Yes. Reflexive thematic analysis (RTA)

3. Is the use and justification of the specific type of TA consistent with the research questions or aims?

Yes. Our aim is to understand how clinicians interpret and navigate complex end-of-life care, an interpretive question

4. Is there a good 'fit' between the theoretical and conceptual underpinnings of the research and the specific type of TA (i.e., is there conceptual coherence)?

Yes. The epistemology section explicitly positions the study within a social constructivist paradigm, compatible with RTA.

5. Is there a good 'fit' between the methods of data collection and the specific type of TA?

Yes. Semi-structured interviews provide thick data for reflexive analysis.

6. Is the specified type of TA consistently enacted throughout the paper?

We have clarified that coding was inductive and interpretive and ensured no positivist language around codebooks, inter-rater reliability, and consensus coding. This is procedurally consistent with RTA.

7. Is there evidence of problematic assumptions about, and practices around, TA?

No. I do not believe so i.e. no references to saturation or objective rating.

8. Are any supplementary procedures or methods justified, and necessary, or could the same results have been achieved simply by using TA more effectively?

Yes. The only supplementary tool, a framework matrix, is described as a data management aid only, not a coding frame.

9. Are the theoretical underpinnings of the use of TA clearly specified?

Yes. We have strengthened the description of our constructivist epistemology, linking it directly to our use of RTA.

10. Do the researchers strive to 'own their perspectives'... their personal and social standpoint and positioning?

Yes. Reflexivity section already present in line with SRPQ reporting.

11. Are the analytic procedures clearly outlined, and described in terms of what the authors actually did?

Response:
Yes. The explicit 6 step list has been removed for clarity and flow but otherwise it is unchanged.

12. Is there evidence of conceptual or procedural confusion?

I do not believe so.

13. Do the authors demonstrate full and coherent understanding of their claimed approach to TA?

Yes. By citing Braun & Clarke (2021), discussing the 20-question tool, and clarifying analytic procedures, I hope the revised manuscript addresses this more fully.

14. Is it clear what and where the themes are in the report?

Response:
Yes. Themes are clearly presented, named, and structured, with supporting narrative and illustrative extracts.

15. Are the reported themes topic summaries rather than fully realised themes, or is this appropriate?

The themes are fully realised patterns of shared meaning with supporting data.

16. Is non-thematic contextualising information presented as a theme?

No. Contextual information is kept separate from themes. Themes are interpretive patterns rather than background description.

17. In applied research, do the themes have potential to give rise to actionable outcomes?

Yes. Themes identify specific challenges and dynamics with implications for end-of-life care practice which is highlighted in the Discussion.

18. Are there conceptual clashes or confusion?

No. I believe there are no errant positivist terms

19. Is there evidence of weak or unconvincing analysis?

I do not believe so. Data analysis was supervised by an experienced qualitative researcher.

20. Do the authors make problematic statements about generalisability?

No.

Findings:
Line 52 under the theme of symptom management. It seems the narrative excerpt should be 'some' instead of 'come'?

Thank you, it should.

Page 11, line 12-13: No symptom assessment described. Was this not a part of the topic guide or if not why was it not further explored? To aid clarity to the sentence here.

I have expanded this sentence to read: Participants did not describe using any formal symptom assessment scales as part of their routine care for patients even on direct questioning, instead relying on a gestalt assessment of patient wellbeing.

We had expected the use of a symptom scale, or even early warning scores/respiratory rates, so it was explored explicitly in the topic guide. Instead participants assessed patient symptoms through unguided clinical assessment.

The study limitations are noted and reflect the location and breadth of the study.

Finally, thank you for bringing professional perspectives to the fore in an end-of-life context and to the inherent complexities involved.

Thank you for your time in reviewing our manuscript. I hope we have been more explicitly transparent with B&C's RTA as a result.

Reviewer: 2
Dr. Marjan Mardani-Hamooleh, Iran University of Medical Sciences
Comments to the Author:
Change the study topic to this form:
How do clinicians navigate end-of-life care with NIV/CPAP? A thematic analysis study

We thank the reviewer for this suggestion. The title has been updated accordingly.

Use references from the last 5 years in the introduction and discussion.

We thank the reviewer for this suggestion. We have reviewed all references and categorised them as follows:

(1) References from within the last 5 years (2020 onwards):
1, 4, 6, 7, 8, 9, 10, 11, 17, 32, 33, 34.

(2) References older than 5 years but methodological, conceptual, or structural (and therefore intentionally retained):
12, 13, 14, 15, 16.

Below, we address each of the remaining references individually, outlining whether a more recent equivalent exists and the changes made where appropriate.

2. Jayadev et al. (2019)
Although slightly older than five years, this remains the most up-to-date peer-reviewed analysis of real-world COPD audit data relating to NIV timing and mortality. No newer study provides equivalent data; therefore, this reference has been retained.

3. Castro-Añón et al. (2015)
This remains the most recent cohort with explicit mortality data for obesity hypoventilation syndrome. We have added Athayde et al. (2018) as a more recent review, but Castro-Añón et al. continues to be the key source for mortality figures in this under-researched area.

5. Cosentini et al. (2008)
We have added a more recent source (Mebazaa et al., 2015) to update the contemporary context. As no newer study provides explicit mortality rates for CPAP in acute cardiogenic pulmonary oedema, we have retained Cosentini et al. alongside the updated citation.

18. Robichaux et al. (2006)
This is a seminal qualitative paper on prognostic conflict and remains without a contemporary peer. It continues to be cited widely and has been retained.

19. Carlet et al. (2004)
To support this older reference, we have added Curtis et al. (2020) regarding decision-making complexities during the COVID-19 pandemic, when patient participation was often impossible. Carlet et al. has been retained as foundational.

20. White et al. (2007)
We have supplemented this reference with Georgakis et al. (2024) to provide updated evidence on shared decision-making and communication in serious illness.

21. Harrington & Smith (2008)
This remains a landmark conceptual paper on overtreatment at the end of life. No modern equivalent supersedes it; therefore, it has been retained.

22. Wright et al. (2010)
This highly influential paper on patient–caregiver outcomes following end-of-life discussions remains without a replacement study of comparable scale. It continues to be widely cited and has been retained.

23. Wendler & Rid (2011)
This systematic review remains the most comprehensive synthesis of surrogate decision-making burdens and is still cited frequently. It has been retained.

24. Seemungal et al. (2009)
This reference has been replaced with the 2024 GOLD Report, which provides updated COPD epidemiology and management guidance (acknowledging that GOLD draws on earlier data where appropriate).

25. Meier et al. (2016)
This remains the most comprehensive and widely cited review of definitions of a "good death." No newer review offers equivalent coverage; therefore, it has been retained.

26. Wright et al. (2008)
Although older than five years, this remains a cornerstone study and continues to be cited in recent literature. No comparable modern replacement exists.

27. Cullati et al. (2019)
This reference is only marginally older than five years and remains current and relevant for team conflict and quality-of-care research. Retained.

28. Puntillo & McAdam (2006)
This foundational reference is now supplemented with Georgakis et al. to provide an updated perspective on clinician–clinician communication.

29. Redelmeier & Tversky (1990)
To ensure currency, this reference has been replaced with more contemporary work by Kaufman (2015) on clinical decision-making and moral reasoning, while preserving the conceptual insight of the original.

30. Allen (2014)
Allen's work represents the original and authoritative articulation of invisible and relational

nursing labour. This is a conceptual, not time-sensitive, reference and has therefore been retained.

31. Nava et al. (2013)
This trial remains the only randomised study of palliative NIV in solid tumours. As no subsequent RCT exists, it continues to be the leading evidence and has been retained.

The introduction is incomplete. Complete the review of previous studies. What was the gap in the previous literature? What was your main concern for doing this work?

We thank the reviewer for this helpful observation. We have substantially revised the final paragraph of the introduction and the aims section to more explicitly articulate the gaps in existing literature, the rationale for the study, and its contribution to current understanding.

The method is flawed. Report the method based on the checklist for qualitative studies. Reference this checklist and write down exactly what it assesses. All subsections of the method are flawed and this section needs serious revision.

We thank the reviewer for highlighting the need for clear reporting standards. We have now made explicit that the study is reported in accordance with the SRQR (Standards for Reporting Qualitative Research) checklist, an internationally recognised guideline for qualitative studies that outlines best practice for reporting study design, researcher reflexivity, data collection, analysis, and trustworthiness. Some subsection titles had previously been revised for readability; these have now been restored to ensure full transparency and alignment with SRQR headings.

VERSION 2 - REVIEW

**Reviewer**	**1**
**Name**	**Jones, Kerry**
**Affiliation**	**The Open University, HWSC WELS**
**Date**	**02-Dec-2025**
**COI**	

Dear authors,

Thank you for addressing the comments in response to the review. I look forward to seeing the paper published in a forthcoming edition and wish you well in future research endeavours.
